# Risk of Feline Immunodeficiency Virus (FIV) Infection in Pet Cats in Australia is Higher in Areas of Lower Socioeconomic Status

**DOI:** 10.3390/ani9090592

**Published:** 2019-08-21

**Authors:** Vivian Tran, Mark Kelman, Michael Ward, Mark Westman

**Affiliations:** 1 Sydney School of Veterinary Science, The University of Sydney, Camperdown, NSW 2006, Australia; 2 Sydney School of Veterinary Science, The University of Sydney, Camden, NSW 2570, Australia

**Keywords:** feline immunodeficiency virus, calicivirus, herpesvirus, socioeconomic, disease, veterinary science, Australia

## Abstract

**Simple Summary:**

Some diseases are known to occur at a higher frequency in Australia in areas of social and economic disadvantage. Identification of these diseases is important for effective infection control strategies. We investigated whether an association existed between socioeconomic factors and three infectious diseases in cats (feline immunodeficiency virus, FIV; feline calicivirus, FCV; and feline herpesvirus-1, FHV-1) in Australia. Disease cases that were reported to a voluntary veterinary disease surveillance system (Disease WatchDog) between January 2010 and July 2017 were extracted and analysed. Postcodes of the owners of these cats were compared to four government-published indexes measuring socioeconomic disadvantage and advantage. An association between socioeconomic status and FIV infection, but not FCV and FHV-1 infection, was found. FIV infection was more commonly reported in areas of socioeconomic disadvantage according to all four indexes. Prevention strategies targeting lower socioeconomic communities may help to reduce the overall prevalence of FIV infection in Australia.

**Abstract:**

Feline immunodeficiency virus (FIV), feline calicivirus (FCV), and feline herpesvirus (FHV-1) are common viral infections of domestic cats in Australia. A study was performed to investigate the possible effect of area-based socioeconomic factors on the occurrence of FIV, FCV, and FHV-1 infection in Australian client-owned cats. A total of 1044 cases, reported to a voluntary Australian online disease surveillance system between January 2010 and July 2017, were analysed with respect to their postcode-related socioeconomic factors using the Socio-Economic Indexes For Areas (SEIFA). SEIFA consists of four different indexes which describe different aspects of socioeconomic advantage and disadvantage. Signalment details including age, sex, neuter status, and breed were also considered. A significant correlation was observed between areas of lower socioeconomic status and a higher number of reported cases of FIV infection for all four SEIFA indexes (*p* ≤ 0.0002). Postcodes with SEIFA indexes below the Australian median (“disadvantaged” areas) were 1.6–2.3 times more likely to have reported cases of FIV infection than postcodes with SEIFA indexes above the median (“advantaged” areas). In contrast, no correlation was observed between the number of reported cases of FCV or FHV-1 infection and any of the four SEIFA indexes (*p* > 0.05). When signalment data were analysed for the three infections, FIV-infected cats were more likely to be older (*p* < 0.00001), male (*p* < 0.0001), neutered (*p* = 0.03), and non-pedigree (*p* < 0.0001) compared to FCV and FHV-1 infected cats. Results from this study suggest that area-based disease control strategies, particularly in areas of social disadvantage, might be effective in reducing the prevalence of FIV infection in pet cats in Australia.

## 1. Introduction

There are currently an estimated 3.3 million pet cats (Felis sylvestris catus) in Australia, with 29% of Australian households owning at least one cat [1,2,3]. Feline immunodeficiency virus (FIV), feline calicivirus (FCV), and feline herpesvirus (FHV-1) are common infections of the domestic cat, both in Australia and overseas, and are major causes of feline morbidity despite the availability of reasonably efficacious vaccines [4,5].

A total of 14.5 million domestic cats and 19 million feral cats worldwide are thought to be infected with FIV [6]. Transmission of FIV is primarily through cat bite wounds, meaning that FIV prevalence is higher where aggression and overcrowding are present, in particular in stray and feral cat populations [7,8]. FIV seroprevalence was reported to be 21% and 25% in two separate feral cat populations in Sydney, Australia, compared to 16% in client-owned cats in eastern Australia with some level of outdoor access [9]. Similarly, in Ottawa, Canada, FIV seroprevalence was found to be 23% in urban stray cats compared to 8% in client-owned cats [10], and an overall FIV seroprevalence in the general cat population of 4% across 10 Canadian provinces has been reported [11]. The disparity in prevalences between unowned cats and client-owned cats in these studies suggests a possible role for stray and feral cats as reservoirs of FIV infection. Another Australian study reported that 80% of client-owned cats had at least some outdoor access, suggesting likely exposure to stray and feral cats and therefore FIV infection [3]. Other known risk factors for FIV infection, apart from lifestyle (i.e., outdoor access and fighting), include increased age (since infection is lifelong and therefore risk of infection is cumulative), sex (male), neuter status (entire), and possibly vaccination status (FIV-unvaccinated) [4,9,12,13,14,15,16,17]. Some studies have found a higher risk of FIV infection in cats classified by veterinarians as clinically “unwell” or “sick”, most likely due to the virus’ predilection for cells of the immune system and subsequent resulting immunosuppression, therefore increasing the risk of opportunistic infections [12,18,19]. Common sequelae of FIV infection include gingivitis, upper respiratory tract infections, toxoplasmosis, and lymphosarcoma [14,20,21,22,23].

FCV and FHV-1 infection are recognised as major causes of feline upper respiratory tract disease (FURTD) worldwide, negatively impacting on feline welfare and quality of life [24,25,26]. FCV and FHV-1 are frequently detected in sick cats in Australia; studies have reported prevalences of 10%–16% for FCV and 7%–21% for FHV-1 in cats presenting with FURTD [27,28,29]. A study of client-owned cats in the US, using a convenience sample of healthy and sick cats combined (i.e., a heterogenous population), reported prevalences of 26% for FCV and 5% for FHV-1, respectively [30]. FURTD is a major cause of euthanasia in shelters, second to overcrowding [31], and stress, overcrowding, and close contact are all important factors in the transmission of both viruses [32,33,34]. One study of eight shelters in the US demonstrated a high variability in prevalences of FCV and FHV-1 infection between shelters studied [31]. In heterogenous shelter populations in Europe and North America, prevalences of 33%–37% for FCV and 11%–20% for FHV-1 have been reported [35,36]. Other known risk factors for FCV and FHV-1 infection include age (kittens and juveniles), sex (male), vaccination status (unvaccinated), and lifestyle (outdoor access and multi-cat households) [30,37]. Due to the viruses’ tropism for upper respiratory and conjunctival epithelium, FCV and FHV-1 infected cats typically present with clinical signs of conjunctivitis, nasal discharge, corneal ulceration, and rhinotracheitis [24,32,33,34,38].

It is widely accepted that humans living in areas of lower socioeconomic status have higher health risks, including infection with human immunodeficiency virus (HIV-1) and human herpesvirus-8 (HHV-8, also known as Kaposi’s sarcoma-associated herpesvirus) [39,40]. Similarly, a study of canine parvovirus (CPV) in client-owned dogs in Australia reported a correlation between areas of socioeconomic disadvantage and CPV infection [41]. To our knowledge, no investigation into a possible relationship between the socioeconomic status of cat owners and feline health has been performed anywhere to date. The aim of the current study, therefore, was to determine if there was an association between the area-based socioeconomic status of cat owners and risk of FIV, FCV, or FHV-1 infection in the pet cat population in Australia.

## 2. Materials and Methods

### 2.1. Study Population

All reported cases of FIV, FCV, and FHV-1 infection were retrieved from the Disease WatchDog database [42]. This disease surveillance system, active between January 2010 and July 2017, relied on veterinary clinics in Australia to voluntarily register online and submit cases of common canine and feline infections.

Extracted cases were screened for duplicates based on patient name and clinic, with the earliest record of the case retained for analysis and the later record excluded. Cases concurrently diagnosed with more than one of the three infections were duplicated and one infection was re-labelled as the secondary disease, ensuring that all disease cases were included in the data analysis but only accounted for once. Details available for each case included patient name, age (years, months, and weeks), sex (male or female), neuter status (neutered, entire, or unknown), breed (pedigree or non-pedigree), primary disease, date of diagnosis, method of diagnosis, and owner postcode. Cases of FIV infection were diagnosed by antibody detection using a rapid in-clinic test (any commercially available brand of test kit) or by polymerase chain reaction (PCR) testing at an external laboratory (IDEXX Laboratories, East Brisbane, Queensland, Australia; or Gribbles Veterinary Pathology, Glenside, South Australia, Australia). Cases of FCV and FHV-1 infection were diagnosed by clinical presentation or by PCR testing at an external laboratory (IDEXX Laboratories, East Brisbane, Queensland, Australia).

### 2.2. Assessment of Area-Based Socioeconomic Status

Socioeconomic data from the 2016 Australian Census was collected by the Australian Bureau of Statistics (ABS) and used by the Australian Government to develop the Socio-Economic Indexes For Areas (SEIFA). SEIFA is a useful tool for interpreting area-based socioeconomic factors and consists of four indexes which describe different aspects of advantage and disadvantage. The indexes are developed in different ways and combine factors including income, education, employment, occupation, and housing variables [43].

The Index of Relative Socioeconomic Advantage and Disadvantage (IRSAD) combines a range of factors that relate to an individual’s social advantage or disadvantage, including education, employment, occupation, housing, language, and disability. The Index of Relative Socioeconomic Disadvantage (IRSD) is similar to the IRSAD except it only examines disadvantage factors, not those of advantage. The Index of Economic Resources (IER) combines factors relating to financial advantage and disadvantage, including high or low income, and rent. The Index of Education and Occupation (IEO) combines education and employment factors relating to social advantage and disadvantage [43].

The ABS uses Census data to assign all Australian postcodes a ranked decile (from 1 to 10) for the four SEIFA indexes (i.e., IRSAD, IRSD, IER, and IEO); 1 represents the most socioeconomically disadvantaged areas and 10 represents the least disadvantaged (i.e., most advantaged) areas. Using this information, cases in the current study of FIV, FCV, and FHV-1 infection were assigned four SEIFA scores by the primary author (VT) based on the recorded owner postcode. We did not assume that cat numbers were equally distributed by SEIFA, since we did not have access to the spatial distribution of cats in Australia during the sampling time period. As far as we are aware, such data are not available, and the spatial distribution of owned cats in Australia remains unknown.

### 2.3. Data Analysis

Chi-squared tests of independence were performed to analyse case signalment (sex, neuter status, and breed) within each infection group (i.e., FIV, FCV, and FHV-1), and between infection groups, using Statistix version 8.0 (Analytical Software, Tallahassee, FL, USA). Statistix was also used to perform Kruskal-Wallis one-way analysis of variance (AOV) testing to compare median ages between the different disease populations. This study did not have a control population, hence there was an expected equal distribution within each binary variable for each disease.

A Spearman’s Rank Correlation test was performed on SEIFA indexes within each infection group (i.e., FIV, FCV, and FHV-1) to measure the strength and association between the two ranked variables (i.e., SEIFA values and number of cases of each infection). Kruskal-Wallis one-way AOV testing was used to compare “disadvantaged” socioeconomic areas (SEIFA indexes below the Australian median score) and “advantaged” socioeconomic areas (SEIFA indexes above the Australian median score) with respect to the number of reported cases of each disease. Odds ratios were calculated from 2 × 2 contingency tables and results of Kruskal-Wallis one-way AOV testing.

Cases of FIV, FCV, and FHV-1 infection were mapped using ArcGIS version 10 (Esri, Redmond, WA, USA). A retrospective space-time analysis scanning for clusters with high rates of disease was performed using SaTScan version 9.4.1 (SaTScan, Boston, MA, USA). Clusters of FIV, FCV, and FHV-1 infection were identified using a maximum spatial cluster size of 5% of the population at risk and a maximum temporal cluster size of 90 days within the study period. A Wilcoxon Rank Sum test was performed to compare SEIFA scores of postcodes within a reported cluster and postcodes outside of clusters using Statistix.

For all analyses, statistical significance was considered to be *p* < 0.05.

## 3. Results

### 3.1. Study Population

Extraction of reported cases of FIV, FCV, and FHV-1 infection from Disease WatchDog yielded 1077 cases from 287 postcodes and 145 veterinary clinics. In total, 37 duplicate case records were removed, and of the remaining 1040 cases, two cats were concurrently infected with FHV-1 and FIV and two cats were concurrently infected with FCV and FHV-1, yielding a final total of 1044 cases for data analysis. FHV-1 was the most commonly reported infection (607/1044, 58%), followed by FCV (256/1044, 25%) and FIV (181/1044, 17%).

Signalment data for each infection is summarised in Table 1. Unfortunately, cases could be submitted to Disease WatchDog with incomplete signalment data. Considering only cases with sex recorded (749/1044, 72%), males were more common than females (461 versus 288, respectively). Of cases with a recorded neuter status (749/1044, 72%), neutered cats were more common than entire cats (490 versus 259, respectively). Of cases with a recorded breed (756/1044, 72%), non-pedigree cats were more common than pedigree cats (585 versus 171, respectively).

FIV infection was most commonly diagnosed by antibody detection (88%), followed by polymerase chain reaction (PCR) testing (12%). FCV and FHV-1 infection were most commonly diagnosed by clinical presentation (92% and 98%, respectively), followed by PCR testing (8% and 2%, respectively; Table 2).

**Figure 1:**
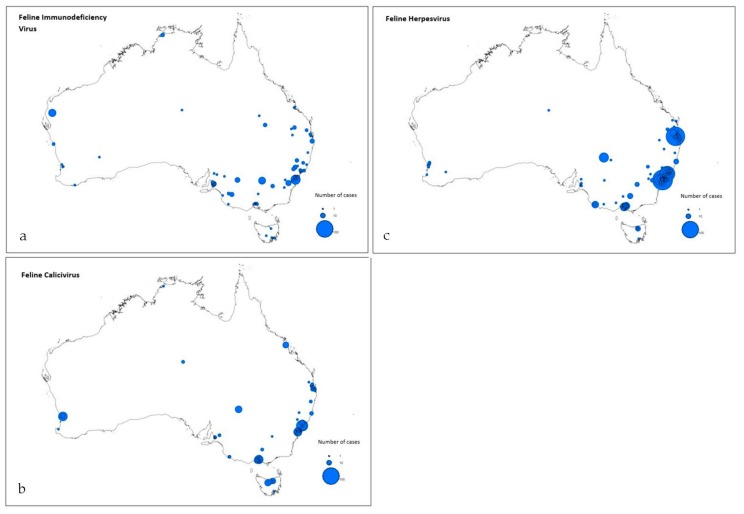
Map of reported cases of (a) feline immunodeficiency virus (FIV), (b) feline calicivirus (FCV), and (c) feline herpesvirus (FHV-1) infection in Australia extracted from Disease WatchDog (2010–2017). Mapped using ArcGIS version 10, Esri, Redmond, WA, USA.

In total, 287 postcodes were reported across Australia, comprising New South Wales (56%), Queensland (16%), Victoria (13%), South Australia (6%), Western Australia (5%), Tasmania (3%), Northern Territory (0.1%), and Australian Capital Territory (0.001%). Mapping of cases for each infection is shown in Figure 1.

### 3.2. Intragroup Analysis

Males were overrepresented in the reported cases of FIV and FCV infection (*p* < 0.0001 and *p* = 0.04, respectively; Chi-squared tests of independence; Table 1). There were significantly more neutered cats than entire cats for FIV, FCV, and FHV-1 infection (*p* < 0.0001 for FIV and FHV-1 infection, *p* = 0.03 for FCV infection; Chi-squared tests of independence) and non-pedigree cases were overrepresented in all three groups (*p* < 0.0001; Chi-squared tests of independence; Table 1).

### 3.3. Intergroup Analysis

When comparing signalment between groups, FIV-infected animals were significantly older than cats with FCV or FHV-1 infection (*p* < 0.00001; Kruskal-Wallis one-way AOV test). Cats with FIV infection had the highest reported median age of 74 months, while cats with FCV or FHV-1 infection had a median age of 12 months and 16 months, respectively (Table 1).

There were significantly more reported cases of FIV-infected males compared to FCV and FHV-1 infected males (82% versus 57% versus 53%; *p* < 0.0001; Chi-squared tests of independence; Table 1). There was also a significant difference in neuter status and breed between groups, with significantly more neutered and non-pedigree cats reported with FIV infection compared to FCV or FHV-1 infection (*p* = 0.025 and *p* < 0.0001, respectively; Chi-squared tests of independence; Table 1).

### 3.4. Risk Factor Analysis

**Figure 2:**
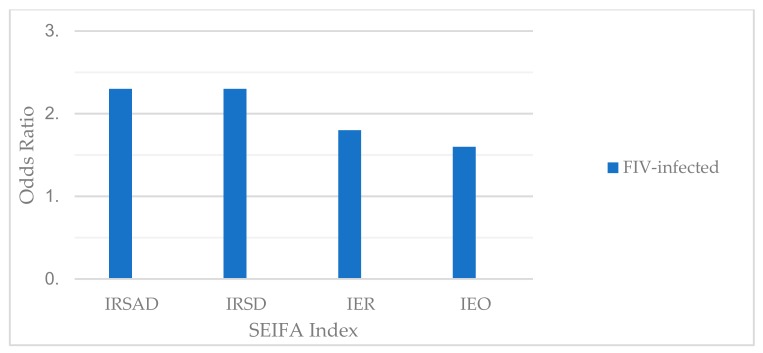
Odds ratios for cases of feline immunodeficiency virus (FIV) infection based on socioeconomic status, calculated by comparing the number of reported cases of FIV infection for “disadvantaged” socioeconomic areas (SEIFA scores below the Australian median score) and “advantaged” socioeconomic areas (SEIFA scores above the Australian median score). Disadvantaged areas were 1.6–2.3 times more likely to have reported cases of FIV infection than advantaged areas (*p* < 0.0001). IRSAD = The Index of Relative Socioeconomic Advantage and Disadvantage. IRSD = The Index of Relative Socioeconomic Disadvantage. IER = The Index of Economic Resources. IEO = The Index of Education and Occupation.

There was a significant negative correlation between reported cases of FIV infection and all four SEIFA indexes, i.e., there was a higher number of FIV cases reported in postcodes with relatively lower SEIFA indexes (*p* = 0.0002; Spearman’s Rank Correlation tests; Table 3). Disadvantaged areas were 1.6–2.3 times more likely to have reported cases of FIV infection than advantaged areas (IRSAD 2.3 times, IRSD 2.3 times, IER 1.8 times, and IEO 1.6 times; *p* = 0.0001; Kruskal-Wallis one-Way AOV tests; Figure 2 and Table 4).

There was no association between reported cases of FCV or FHV-1 infection and any of the four SEIFA indexes (*p* > 0.05; Spearman’s Rank Correlation tests; Table 3), nor any difference when disadvantaged and advantaged areas were compared (*p* > 0.05; Kruskal-Wallis one-way AOV tests; Table 4).

### 3.5. Cluster Analysis

There were seven, two, and eleven space-time disease clusters of FIV, FCV, and FHV-1 cases identified, respectively. When the median scores of SEIFA indexes for clustered and non-clustered postcodes were compared, a significant difference was observed for three of the four SEIFA indexes for FIV and FCV infection and all four SEIFA indexes for FHV-1 infection (*p* < 0.0001; Wilcoxon Rank Sum test; Table 5). In all analyses, the median SEIFA score was higher for clusters than for non-clusters, suggesting that clusters of case reports occurred in areas of socioeconomic advantage.

## 4. Discussion

We conducted an exploratory, hypothesis-generating study using a voluntary case reporting disease surveillance system. Cats with FIV infection were 1.6–2.3 times more likely to reside in disadvantaged areas (socioeconomic scores lower than the median) than advantaged areas, demonstrating a possible connection between socioeconomic status and feline health in Australia. Comparable findings have been reported in dogs in Australia, with CPV cases more likely to occur in areas of greater relative socioeconomic disadvantage [41], and dogs in communities with lower economic status more likely to be infected with zoonotic pathogens, parasites, and canine distemper virus [44]. Other studies have reported correlations between relative lower income and education with canine obesity, as well as reduced knowledge of pet healthcare and reduced inclination to spend money on preventive pet healthcare interventions such as vaccination [45,46,47,48]. Recently, a global study of FIV prevalences reported decreasing FIV infection with increasing national income, suggesting a possible causal relationship between the investment in animal control programs and subsequent impact on FIV prevalence [49]. Despite the currently available FIV vaccine (Fel-O-Vax FIV^®^, Boehringer Animal Health, Fort Dodge, IA, USA) only having a published protective rate in Australia of 56% [4], it is also possible that a lower vaccine uptake in disadvantaged areas of Australia compared to advantaged areas might have contributed to the higher number of FIV cases reported in these areas in the current study. Despite FIV vaccination causing a diagnostic challenge for cases of FIV infection, with production of antibodies following FIV vaccination indistinguishable from antibody production following natural infection using some in-clinic antibody test kits [50], we considered it highly unlikely that FIV-vaccinated cats were incorrectly diagnosed with FIV infection and reported to Disease WatchDog since this cross-reaction is well known by Australian veterinarians and complete vaccination histories for client-owned cats are usually available. Whether the FIV vaccination rate plays a role in clusters of case reports is unknown since companion animal vaccination rates by geographic area is currently not reported or published. Regardless, this finding suggests that consideration needs to be given to providing FIV vaccination as a part of community pet health programs, particularly when FIV “outbreaks” are suspected by local veterinarians.

In contrast to FIV infection, no correlation was observed between cases of FCV and FHV-1 infection and owner socioeconomic status. This difference may be due to vigilant vaccination against these diseases in Australia, as recommended in the World Small Animal Veterinary Association and Australian Veterinary Association vaccination guidelines [51,52]. Vaccination rates in Australia for FCV and FHV-1 are likely very high, with a survey of Australian and New Zealand pet owners reporting that 88% believed regular vaccinations were necessary [2].

There was evidence of case clustering for FIV, FCV, and FHV-1 infection in socioeconomically advantaged areas. This finding may have been a reporting phenomenon, due to a higher concentration of clinics registered with Disease WatchDog in urban areas, which are generally associated with higher SEIFA scores compared to all postcodes throughout Australia. In addition, with regards to FCV and FHV-1 infection (the major causes of FURTD), the observed association between disease clustering and socioeconomic advantage may reflect risk factors related to the behaviour of higher socioeconomically ranked owners. For example, cat owners who were more likely to board their cats in a cattery during holiday periods, with resulting increased exposure to FURTD, or reactivation of latent FHV-1 virus due to boarding-associated stress.

Age (older cats) was found to correlate with increased risk of FIV infection, presumably due to the lifelong nature of infection. Non-pedigree and neutered cats were overrepresented with FIV, FCV, and FHV-1 infection, likely due to a sampling bias. One study of pet cats in Australia and New Zealand reported that 72% were non-pedigree cats and 94% were neutered [2]. Male cats were at increased risk of FIV and FCV infection, but not FHV-1 infection. It is known that male cats are at higher risk of FIV infection due to increased territorial behaviour and fighting [7,12]. The explanation for the FCV and FHV-1 findings is unknown, especially since a previous Australian study found that male cats were at increased risk of FHV-1 (but not FCV) infection [53].

The main constraint of the current study was the source of the sample population. Due to the voluntary nature of clinical case submissions by veterinarians, the total number of cases of each infection in Australia could not be estimated, nor prevalence for each infection determined. We were limited in the risk factors that could be explored, and it is possible that the cases extracted and analysed were not a representative sample for the period selected. The number of clinics registered with and submitting cases to Disease WatchDog varied from 10% (222/2260) of companion animal veterinary hospitals in Australia at its peak in 2010, to 3% (58/2260) at the close of Disease WatchDog in 2017 [54,55], and it was not possible to determine retrospectively the proportion of registered clinics that were located in areas of social disadvantage compared to areas of social advantage. Another limitation of the study was a lack of identification in the Disease WatchDog data as to the ownership status of the cat (e.g., whether the cat was privately owned, residing in a shelter, or feral). It was assumed that most cases were owned cats due to private veterinary clinics submitting cases, thereby creating a bias towards owned cats and away from stray and feral cats. Some clinics, however, could have been performing shelter work or treating stray cats, and it would be useful in future studies to record the ownership status of the cat to enable reporting of the frequency of each infection in different cat populations. There was also a potential limitation using SEIFA indexes in the absence of spatial data regarding the distribution of cats in Australia, and the reported differences in the current study could be explained by a non-uniform distribution of cats by SEIFA if it is assumed that FIV infection is uniformly distributed across Australia. Finally, reliance on clinical signs rather than molecular diagnostics in the majority of FURTD cases to diagnose FCV and FHV-1 infections may have led to some errors in case assignment. We note that different aspects of FCV and FHV-1 infection from the current dataset have previously been published in the peer-reviewed literature [29,53]. Because of this reliance on clinical signs for the diagnosis of most cases of FURTD in the current study, we understated the importance of the observed difference in case signalment (i.e., males at apparent greater risk of FCV infection) and recommend future studies to limit case recruitment to those with appropriate diagnostic testing performed.

## 5. Conclusions

The results of area-based risk factor analysis demonstrated an association between cases of FIV infection and socioeconomic disadvantage in Australia. Area-targeted FIV vaccination and education programs might help reduce the incidence of FIV-associated disease. The contagious nature of FIV, FCV, and FHV-1 infection can cause clusters of disease in time and geographical space, and in the current study, clusters of case reports for each infection occurred in socioeconomically advantaged areas in Australia. Timely identification of clusters may lead to the development of targeted disease prevention strategies and permit further epidemiological investigation.

## Figures and Tables

**Table 1:** Signalment data for reported cases of feline immunodeficiency virus (FIV), feline calicivirus (FCV), and feline herpesvirus (FHV-1) infection in Australia between 2010 and 2017, extracted from Disease WatchDog. Results from Chi-squared independence testing within each infection group (intragroup analysis) are displayed in brackets. Results from Chi-squared independence testing comparing infection groups (intergroup analysis) are reported in the text.

Disease	No. of Cases	Median Age and Range	Sex Distribution (M *vs.* F)	Neutering Status (Entire *vs.* Neutered)	Breed (Pedigree *vs.* Non-Pedigree)
FIV	181	74 months (5–204)	82% *vs.* 18% (*p* < 0.0001)	28.5% *vs.* 71.5% (*p* < 0.0001)	5.5% *vs.* 94.5% (*p* < 0.0001)
FCV	256	12 months (0.3–216)	57% *vs.* 43% (*p* = 0.04)	41.5% *vs.* 58.5% (*p* = 0.03)	33% *vs.* 67% (*p* < 0.0001)
FHV-1	607	16 months (0.5–240)	53% *vs.* 47% (*p* = 0.2)	33.5% *vs.* 66.5% (*p* < 0.0001)	25% *vs.* 75% (*p* < 0.0001)

**Table 2:** Methods of diagnosis for reported cases of feline immunodeficiency virus (FIV), feline calicivirus (FCV), and feline herpesvirus (FHV-1) infection in Australia between 2010 and 2017, extracted from Disease WatchDog.

Disease	Method of Diagnosis				Total
	Clinical Presentation	In-House Antibody Testing	PCR Testing	Not Recorded	
FIV	NA	153	21	7	181
FCV	230	NA	21	5	256
FHV-1	592	NA	14	1	607
Total	822	153	56	13	1044

NA = not applicable. PCR = polymerase chain reaction.

**Table 3:** Comparison of the number of reported cases of feline immunodeficiency virus (FIV), feline calicivirus (FCV), and feline herpesvirus (FHV-1) infection and postcode SEIFA indexes ranked 1 to 10, using Spearman’s Rank Correlation testing. A negative number indicates a negative correlation, i.e., there were fewer cases reported as the SEIFA index rank increased (more advantaged). A positive number indicates positive correlation, i.e., there were more cases reported from areas with higher SEIFA indexes. Significance was considered at *p* < 0.05.

Disease	IRSAD	*p* Value	IRSD	*p* Value	IER	*p* Value	IEO	*p* Value
FIV	−0.0740	0.0002	−0.0777	0.0001	−0.0847	<0.0001	−0.0826	<0.0001
FCV	−0.0069	0.73	−0.0169	0.40	−0.0187	−0.35	−0.0173	0.39
FHV-1	0.0174	0.39	0.0012	0.95	−0.0232	0.35	−0.0056	0.78

IRSAD = The Index of Relative Socioeconomic Advantage and Disadvantage. IRSD = The Index of Relative Socioeconomic Disadvantage. IER = The Index of Economic Resources. IEO = The Index of Education and Occupation.

**Table 4:** Comparison of number of reported cases of feline immunodeficiency virus (FIV), feline calicivirus (FCV) and feline herpesvirus (FHV-1) infection between “disadvantaged” areas (SEIFA index less than the Australian median score) and “advantaged” areas (SEIFA index more than the Australian median score), using Kruskal-Wallis one-way analysis of variance testing. Significance was considered at *p* < 0.05.

Disease	IRSAD	*p* Value	IRSD	*p* Value	IER	*p* Value	IEO	*p* Value
FIV	14.77	0.0001	20.21	<0.0001	14.78	0.0001	18.21	<0.0001
FCV	0.12	0.73	0.91	0.34	0.55	0.46	1.94	0.16
FHV-1	0.79	0.37	0.01	0.91	0.63	0.43	0.17	0.68

IRSAD = The Index of Relative Socioeconomic Advantage and Disadvantage. IRSD = The Index of Relative Socioeconomic Disadvantage. IER = The Index of Economic Resources. IEO = The Index of Education and Occupation. SEIFA = Socio-Economic Indexes for Areas.

**Table 5:** Wilcoxon Rank Sum test mean ranks on disease cluster (C) and non-cluster (NC) postcodes identified using space-time permutation modeling from cases reported in Disease WatchDog from January 2010 to July 2017. Significance was considered at *p* < 0.05.

Disease	IRSAD			IRSD			IER			IEO		
	NC	C	*p*	NC	C	*p*	NC	C	*p*	NC	C	*p*
FIV	1211.5	1582.9	<0.0001	1216.5	1525.0	<0.0001	1248.6	1153.4	0.072	1198.7	1737.4	<0.0001
FCV	1213.8	1915.6	<0.0001	1220.7	1745.0	<0.0001	1241.5	1228.0	0.86	1207.6	2084.5	<0.0001
FHV-1	1203.8	1455.9	<0.0001	1205.3	1447.1	<0.0001	1215.5	1388.1	<0.0001	1205.9	1446.8	<0.0001

IRSAD = The Index of Relative Socioeconomic Advantage and Disadvantage. IRSD = The Index of Relative Socioeconomic Disadvantage. IER = The Index of Economic Resources. IEO = The Index of Education and Occupation.
